# Supplementary material for: Deterministic early endosomal maturations emerge from a stochastic trigger-and-convert mechanism
Source: Nat Commun. 2023 Aug 2;14:4652. doi: 10.1038/s41467-023-40428-1 (PMC10397212; doi:10.1038/s41467-023-40428-1)
Supplement: Supplementary file 3 — Description of Additional Supplementary Files [file 41467_2023_40428_MOESM3_ESM.pdf]

Description of Additional Supplementary Files

File Name: Supplementary Movie 1
Description: Representative whole-cell LLSM imaging of APPL1 and EEA1.

File Name: Supplementary Movie 2
Description: APPL1-EEA1 tracking analysis of whole-cell imaging.

File Name: Supplementary Movie 3
Description: Representative single APPL1–EEA1 endosome collision to conversion

File Name: Supplementary Movie 4
Description: Nocodazole inhibits endosomal conversion.

File Name: Supplementary Movie 5
Description: Visualising dynamic APPL1–EEA1 counter-clustering using SRRF.

File Name: Supplementary Movie 6
Description: N-terminal precedes C-terminal EEA1 binding on converting endosomes.

File Name: Supplementary Movie 7
Description: Representative EEA1 N-terminal to C-terminal conversion with intensity trace.

File Name: Supplementary Movie 8
Description: EEA1 N-terminal is required for endosomal conversions.

File Name: Supplementary Movie 9
Description: Simulating conversion phenomenology using agent-node based modelling.
