# Supplementary material for: Deterministic early endosomal maturations emerge from a stochastic trigger-and-convert mechanism
Source: Nat Commun. 2023 Aug 2;14:4652. doi: 10.1038/s41467-023-40428-1 (PMC10397212; doi:10.1038/s41467-023-40428-1)
Supplement: Supplementary file 13 — Reporting Summary [file 41467_2023_40428_MOESM13_ESM.pdf]

nature portfolio

Corresponding author(s): Harrison York, Srividya Iyer-Biswas, Senthil Arumugam

Last updated by author(s): 13/7/23

Reporting Summary

Nature Portfolio wishes to improve the reproducibility of the work that we publish. This form provides structure for consistency and transparency in reporting. For further information on Nature Portfolio policies, see our Editorial Policies and the Editorial Policy Checklist.

Statistics

For all statistical analyses, confirm that the following items are present in the figure legend, table legend, main text, or Methods section.

n/a	Confirmed	
☐	☒	The exact sample size (n) for each experimental group/condition, given as a discrete number and unit of measurement
☐	☒	A statement on whether measurements were taken from distinct samples or whether the same sample was measured repeatedly
☐	☒	The statistical test(s) used AND whether they are one- or two-sided<br>*Only common tests should be described solely by name; describe more complex techniques in the Methods section.*
☒	☐	A description of all covariates tested
☒	☐	A description of any assumptions or corrections, such as tests of normality and adjustment for multiple comparisons
☐	☒	A full description of the statistical parameters including central tendency (e.g. means) or other basic estimates (e.g. regression coefficient) AND variation (e.g. standard deviation) or associated estimates of uncertainty (e.g. confidence intervals)
☐	☒	For null hypothesis testing, the test statistic (e.g. F, t, r) with confidence intervals, effect sizes, degrees of freedom and P value noted<br>*Give P values as exact values whenever suitable.*
☒	☐	For Bayesian analysis, information on the choice of priors and Markov chain Monte Carlo settings
☒	☐	For hierarchical and complex designs, identification of the appropriate level for tests and full reporting of outcomes
☒	☐	Estimates of effect sizes (e.g. Cohen's d, Pearson's r), indicating how they were calculated

Our web collection on statistics for biologists contains articles on many of the points above.

Software and code

Policy information about availability of computer code

Data collection	Commercial software used: 3i Slidebook, LabView, Nikon Elements, Leica Falcon, NumPy, FiJi.
Data analysis	MATLAB, OriginLab, Custom Python tracking code.

For manuscripts utilizing custom algorithms or software that are central to the research but not yet described in published literature, software must be made available to editors and reviewers. We strongly encourage code deposition in a community repository (e.g. GitHub). See the Nature Portfolio guidelines for submitting code & software for further information.

Data

Policy information about availability of data

All manuscripts must include a data availability statement. This statement should provide the following information, where applicable:

- Accession codes, unique identifiers, or web links for publicly available datasets
- A description of any restrictions on data availability
- For clinical datasets or third party data, please ensure that the statement adheres to our policy

Owing to the large size of the data associated with this manuscript (<70 TB), data will be made available upon request.

Research involving human participants, their data, or biological material

Policy information about studies with human participants or human data. See also policy information about sex, gender (identity/presentation), and sexual orientation and race, ethnicity and racism.

Reporting on sex and gender	Not applicable
Reporting on race, ethnicity, or other socially relevant groupings	Not applicable
Population characteristics	Not applicable
Recruitment	Not applicable
Ethics oversight	Not applicable

Note that full information on the approval of the study protocol must also be provided in the manuscript.

Field-specific reporting

Please select the one below that is the best fit for your research. If you are not sure, read the appropriate sections before making your selection.

[x] Life sciences [ ] Behavioural & social sciences [ ] Ecological, evolutionary & environmental sciences

For a reference copy of the document with all sections, see nature.com/documents/nr-reporting-summary-flat.pdf

Life sciences study design

All studies must disclose on these points even when the disclosure is negative.

Sample size	No statistical method was used to predetermine sample size. Sample size were chosen based on statistical evaluation of the data post acquisition and strength of effect observed. For all the microscopy based assays, each repeat included sufficient cell numbers to ensure that the effects were robustly measured and was repeated at least 3 times. For live cell dynamic measurements, >1000endosomes were observed for each experiment and were repeated more than 5 times.
Data exclusions	Data exclusions were not pre-established. In the LLSM experiments, Samples with cells that appeared abnormal/ unhealthy were excluded to have consistent quality of cells across the measurements.
Replication	All attempts at replication of data contained in this manuscript were successful. Experiments were performed at least three times unless otherwise noted. The crucial experiments were repeated by two independent researchers - SA and HY across a two year period.
Randomization	No method of randomization is applicable here as this study used cultured cell lines which are homogeneous and isogenic.
Blinding	Experiments are not blinded because the experiments and analysis were performed by the authors, and it was not possible to analyze datasets blindly. Key experiments were performed by 3 independent researchers. To reduce potential human bias, quantitative measurements were performed using custom automated image processing algorithms (codes available on https://github.com/EndoMAT. For live cell imaging in LLSM, cells were selected before their behavior was known.

Reporting for specific materials, systems and methods

We require information from authors about some types of materials, experimental systems and methods used in many studies. Here, indicate whether each material, system or method listed is relevant to your study. If you are not sure if a list item applies to your research, read the appropriate section before selecting a response.

Materials & experimental systems

n/a	Involved in the study	
[ ]	[x]	Antibodies
[ ]	[x]	Eukaryotic cell lines
[x]	[ ]	Palaeontology and archaeology
[x]	[ ]	Animals and other organisms
[x]	[ ]	Clinical data
[x]	[ ]	Dual use research of concern
[x]	[ ]	Plants

Methods

n/a	Involved in the study	
[x]	[ ]	ChIP-seq
[x]	[ ]	Flow cytometry
[x]	[ ]	MRI-based neuroimaging

Antibodies

Antibodies used	71-7500, Invitrogen (GST Primary antibody) at 1:500 dilution<br>A-21245 Thermofisher scientificGoat anti-Rabbit AlexaFluor647 secondary antibody at 1:500 dilution
Validation	Antibodies were validated by control cells not expressing GST-FYVE plasmids.

Eukaryotic cell lines

Policy information about cell lines and Sex and Gender in Research

Cell line source(s)	hTERT RPE-1 , sourced from ATCC, WT HeLa and EEA1 KO HeLa souced from Marino Zerial's lab at Max Planck Institute for Cell Biology and Genetics, Dresden, Germany.
Authentication	Authenticated by Monash genome modification facility - genotyping service.
Mycoplasma contamination	All cell lines tested negative for Mycoplasma contamination.
Commonly misidentified lines<br>(See ICLAC register)	HeLa cells, which was authenticated before use. EEA1 KO cell lines from Zerial lab, MPI-Dresden were also authenticated.
